# Ultrasonography as a non‐invasive technique to assess the effects of diet on the ovaries of female European seabass (*Dicentrarchus labrax*)

**DOI:** 10.1111/jfb.70406

**Published:** 2026-04-15

**Authors:** Joaquim Tomàs‐Ferrer, Irene Moro‐Martínez, Enrique Massutí‐Pascual, Amàlia Grau, Miquel Palmer

**Affiliations:** ^1^ LIMIA‐IRFAP, IRFAP, CAIB Unitat Associada al CSIC per l'IMEDEA Port d'Andratx Spain; ^2^ Institut Mediterrani d'Estudis Avançats (IMEDEA), UIB‐CSIC Esporles Spain; ^3^ Institut d'Investigacions Agroambientals i d'Economia de l'Aigua (INAGEA), UIB‐INIA‐CAIB Palma Spain

**Keywords:** bioenergetic models, individual variability, ovaries, reproductive dynamics, ultrasonography

## Abstract

Broodstock management in aquaculture aims to optimise larval production to meet farm demand, which requires precise monitoring of the reproductive cycle. Traditional methods such as histology often require killing of fish, making them unsuitable for monitoring reproductive dynamics at fish level. This study evaluates ultrasonography as a non‐invasive tool for describing ovary size dynamics in European seabass (*Dicentrarchus labrax*). We monitored 48 females fed with different feed amounts with ultrasounds along an entire reproductive cycle, to assess the effect of feed intake on ovarian development. The ovary volume estimated with ultrasounds has been found to be highly correlated with ovary weight (*r* ^2^ = 0.94, *n* = 36). The temporal dynamics of ovary size were modelled using a Bayesian approach, revealing relevant between‐individual variability in maximum ovary weight, timing and duration of ovarian development. Results indicate that individual, but not cage‐level, food intake significantly affects maximum ovary size [slope = 11.12 ovary g/pellets per meal; 95% confidence interval (CI) between 5.29 and 16.81], although it has negligible effects on the date at which the maximum is reached and on the duration of the ovarian cycle. These findings evidence that ultrasonography can be used for an effective monitoring of ovary size dynamics in European seabass, providing real‐time insights for broodstock management with minimal harming to fish. Precise estimates of the maximum ovary size, the date in which this maximum is reached and the ovarian cycle duration may help to identify the best candidate females for covering farm larval demand at any moment, thus enhancing welfare and broodstock management efficiency.

## 1 INTRODUCTION

Broodstock management in aquaculture is aimed to provide enough larvae to ensure production for grow‐out farms (Berlinsky et al., 2020). However, the gametogenic cycle is a complex process driven by the hypothalamus–pituitary–gonad axis (Nagahama et al., 1994), which acts as the main endocrine pathway integrating multiple internal and external factors, including environmental cues (e.g., photoperiod and temperature), social factors (e.g., size structure, dominance/hierarchy) and physiological conditions (e.g., adequate nutritional status) (Migaud et al., 2013). Therefore, effective management plans for controlling the spawning process and ensuring sufficient and continuous gamete production can be designed only when all these cues are well understood, a goal currently achieved for a few species only (Teletchea & Fontaine, 2014). Moreover, accurate predictions of ovulation, crucial for a fine tuning of larval production, require detailed monitoring of the gonadal development as a reliable proxy of the reproductive status of broodfish (Boles et al., 2022; Ortenburger et al., 1996).

Histological examination of gonad tissues (Estay et al., 2003; Taranger et al., 1999), measurement of gonadosomatic index (Mlingi et al., 2023), analysis of blood sex hormone levels (King & Pankhurst, 2003; Mañanós et al., 1997; Taranger et al., 1998), gonad biopsy (Alvarez‐Lajonchère et al., 2001) and gene expression analysis (Díaz & Piferrer, 2015) are standard benchmarks for understanding the gametogenic cycle in aquaculture. However, they may not be reliable techniques for routinely monitoring broodstock and updating management plans. Gonad dissection, for estimating gonad size, describing its histological state or analysing gene expression, requires killing of the fish. These methods are therefore not suitable for individual monitoring (Breton & Berlinsky, 2014; Bureau du Colombier et al., 2015) because they provide only a static snapshot of information, implying that several individuals must be euthanised over time to monitor the group‐level reproductive cycle (Næve et al., 2018). Although ovary biopsy for oocyte collection and blood sample analysis for hormonal profiling are less invasive, they can stress mature broodfish, introduce pathogens, delay or prevent ovulation and even pose a risk to the survival of examined fish (Blythe et al., 1994; Eriksen et al., 2015; Swenson et al., 2007). These limitations underscore the need to explore less invasive techniques that offer a continuous monitoring capability of individual fish.

Ultrasonography has become a promising non‐invasive tool for monitoring the size of internal organs in fishes, particularly gonads (Jennings et al., 2005; McGarvey et al., 2021; Næve et al., 2018). By offering real‐time insights, ultrasonography enables continuous monitoring of gonad morphological dynamics as a possible approach for optimising breeding strategies in aquaculture management. Ultrasonography has been already successfully applied in many fish species, such as European seabass (Macrì et al., 2013), striped bass (Jennings et al., 2005), salmonids (Næve et al., 2018), rainbow trout (Evans et al., 2004), eels (Bureau du Colombier et al., 2015; Jéhannet et al., 2017), sturgeons (Bonev & Nikolova, 2019; Bryan et al., 2007; Petochi et al., 2011), lumpfish (Mlingi et al., 2023) and *Prochilodus brevis* (Salmito‐Vanderley et al., 2023).

In addition, ultrasonography can assess a large number of specimens, which allows us to explore between‐fish variability in the dynamics of ovary development. Although group‐level data may describe the overarching trends, assessing between‐individual variability is paramount for making precise management decisions. This capability becomes particularly relevant for the cases in which the assessment of the gametogenic cycle of certain female broodfish (e.g., early or late spawners) is needed for fulfilling aquaculture requirements for egg and larval production (Migaud et al., 2013).

Individual variability is a ubiquitous property of any biological system (Des Roches et al., 2018). Between‐fish variability in the phenology of the gametogenic cycle is well known (Asturiano et al., 2002; Slesinger et al., 2021). Such variability is related not only to intrinsic differences, for example, genetic factors (Liu et al., 2017; Roa et al., 2008), but also to specific environmental conditions experienced throughout the fish's life span, for example, photoperiod and temperature conditions (Kjesbu et al., 2010; Migaud et al., 2010). Some bioenergetic models, such as the ones that emerged from the Dynamic Energy Budget (DEB) theory (Kooijman, 2010), explicitly consider the processes of energy assimilation and allocation to either growth or reproduction. According to these models, the gametogenic cycle and ovary size dynamics are expected to be regulated by the energetic status of an individual (Muller et al., 2019). From the experimental side, nutrition seems to affect fish reproduction at many levels, including gametogenesis and spawning (Berlinsky et al., 2020). Moreover, egg production has been suggested to depend not only on female size but also on its energetic status and feeding capacity (Serrat et al., 2019). Therefore, broodstock nutrition is expected to play a vital role in determining the spawning success (Migaud et al., 2013).

The European seabass (*Dicentrarchus labrax*) was selected as the model species due to its central role in Mediterranean aquaculture and its suitability for controlled experimental research. Its domestication has made this species relatively easy to handle and maintain under standardised conditions. *D. labrax* is a eurythermal marine fish that inhabits shallow coastal waters from the North‐Eastern Atlantic to the Mediterranean and the Black Sea. It is a dioic species (i.e., with distinct unisexual individuals, either male or female). First sexual maturity in Mediterranean populations occurs at 2–3 years of age for males and 3–4 years of age for females (Pérez‐Ruzafa & Marcos, 2014). It is a synchronous gonochoric species, with spawning taking place once a year during winter (Asturiano et al., 2000; Fritsch et al., 2007; Pawson et al., 2007). Reproduction season in natural conditions extends from September to March in the Mediterranean, when temperatures range between 11 and 15°C (Barnabe et al., 1976; Mañanós et al., 1997). The European seabass is of major commercial importance: aquaculture accounts for 96% of its total production (FAO, 2016), and since the 1980s, it has been one of the most widely farmed species throughout the Mediterranean basin (Chatain & Chavanne, 2009; Vandeputte et al., 2019).

Accordingly, the aims of this contribution are (1) to describe and validate a protocol for estimating ovary size by ultrasonography, (2) to describe between‐fish variability in the ovary size dynamics (peak and spread) throughout the annual gametogenic cycle and (3) to test the effects of feed intake on the ovary size dynamics.

## 2 MATERIALS AND METHODS

### 2.1 Fish husbandry and experimental design

All the *D. labrax* specimens monitored were supplied by an aquaculture company (Aquicultura Balear S.A.U.) located at Palma (Balearic Islands) and were transported as adults to the IRFAP‐LIMIA facilities at Port d'Andratx (Balearic Islands). A total of 48 *D. labrax* females were individually tagged with a passive integrated transponder. At the start of the experiment, the fish weighed 2469 ± 365 g, measured 58.1 ± 3.1 cm of total length (mean ± SD) and were 4 years old. The specimens were randomly distributed in groups of eight females in six different sea cages of approximately 8 m^3^ each, located at 200 m from the shoreline, where the water temperature fluctuates annually between 11.0 and 29.8°C. In addition, two male specimens were added to each cage before the start of the experiment to promote normal ovarian development in females, which seems to be also triggered by male presence via social and chemical cues (Migaud et al., 2013). Males were in the cage but isolated from the females by a second net to preserve the feeding dynamics previously quantified for each group of females (see the following paragraph).

Fish were fed with a dry pellets commercial diet for seabass marine broodstock (Vitalis REPRO, Skretting). Feed pellets had an average weight of 0.701 ± 0.02 g, a digestible energy of 19.1 kJ/g and a 9% water content. The amount of feed delivered per cage and meal was periodically re‐adjusted as a function of temperature and biomass, following the manufacturer's guidelines. However, it is well known that a group of similar‐sized fish reared in a same cage can largely differ in the quantity of feed consumed (Azaza et al., 2013). Therefore, to promote competition for food and enhance between‐fish variability, three levels of food were delivered between cages, with two cages at each level, which were 60%, 75% and 90% of the amount recommended by the manufacturer (referred thereafter as *low*, *medium* and *high* diet). These levels were set since May 2021 (6 months before any reproductive activity). Individual pellet mean consumption had been quantified previously in a parallel experiment, in which individual feed intake rate was video‐monitored using an automated feeder equipped with an underwater camera. In this experiment, the number of pellets consumed by each fish was assessed across eight replicated feeding trials per cage. These feeding assessments were conducted with the same fish groups, at the same cages and under the same social conditions. We found small within‐fish variability: the same fish consistently consumed a similar number of pellets across several monitored meals, compared with larger between‐fish differences. Similar findings were reported by Batzina et al. (2020). Therefore, in accordance with the aims of the study, the average number of feed pellets consumed per meal by the *i* fish (*intake*_*i*_) was standardised by the amount of feed pellets delivered per meal at the cage level and was used as an explanatory variable for modelling ovarian dynamics (see Section 2.4).

### 2.2 Ultrasonography

A total of 369 ultrasound analyses were completed between November 2021 and April 2022, fully covering the species spawning season (Pawson et al., 2007). The 48 fish underwent ultrasound examinations at nominal 14‐day intervals, with minor deviations due to weather‐related constraints. The number of ultrasound analyses per fish ranged between 2 and 12, provided that 36 of the 48 individuals were euthanised sequentially throughout the span of the experiment, to dissect the ovaries and calibrate the ultrasound measurements against the actual ovary weight; consequently, fish euthanised earlier contributed with fewer measurements, whereas the 12 individuals kept alive until the end of the experiment contributed with the maximum number of observations. The 36 fish euthanised were euthanised with an overdose of buffered anaesthetic tricaine methanesulfonate (MS‐222) [dose of 500 mg/L for at least 20 min, as in Topic Popovic et al. (2012)]. The remaining 12 fish were kept alive during the whole experiment.

**FIGURE 1:**
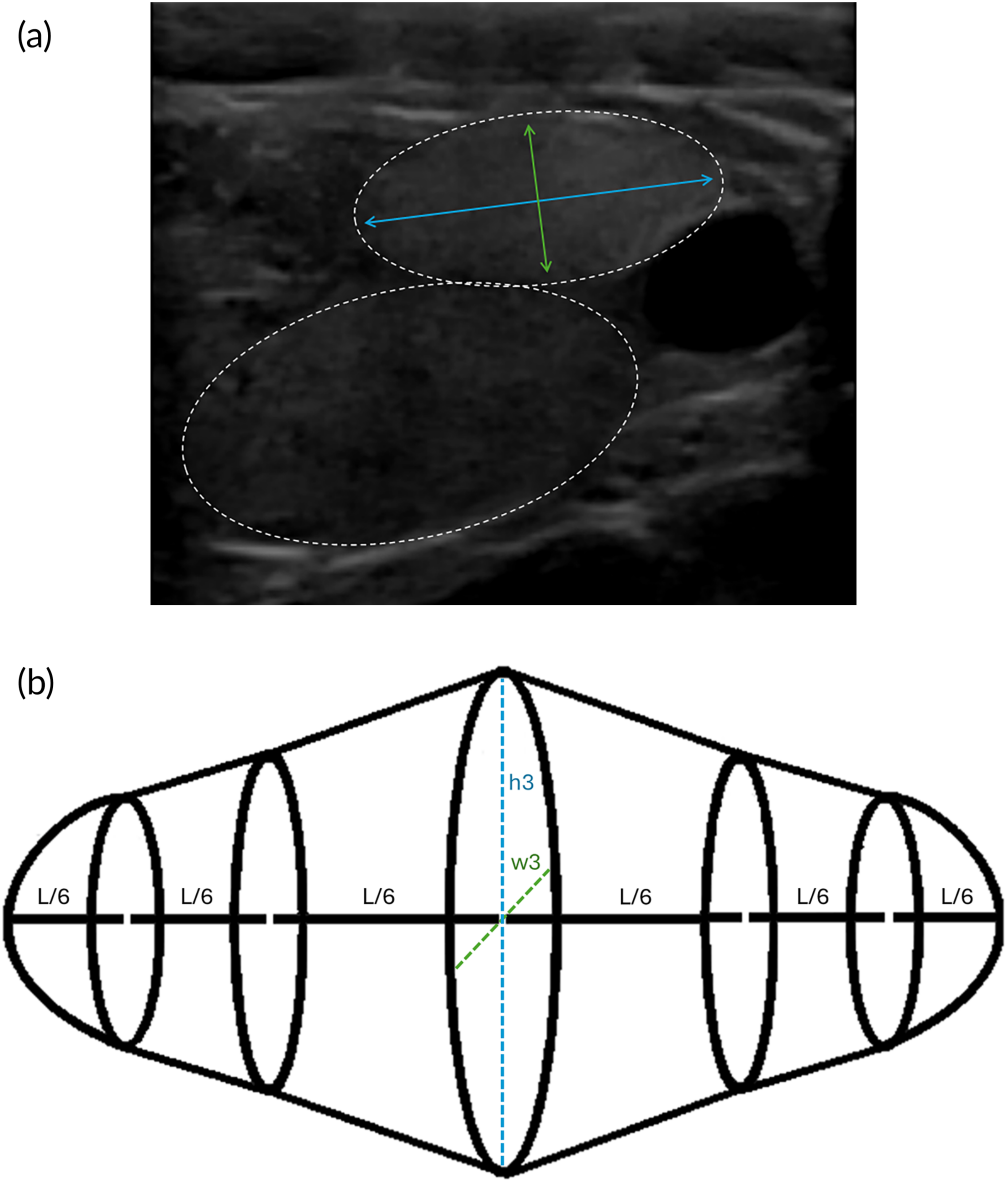
(a) Example of an ultrasound image (transversal plane), showing the two ovary lobes and the height (blue) and width (green) measurements on the left lobe; (b) generic geometrical shape construction used to calculate ovary lobe volume from ultrasound measurements. Height and width measurements are represented in the central slice. The distance between each slice or slice and ovary end is one‐sixth of the ovary lobe total length.

The volume of an organ can be determined from ultrasound measurements by approximating the organ's shape using a combination of simpler geometrical shapes (Bryan et al., 2007). Only the left ovary lobe was measured to estimate the ovary volume. This simplification was adopted to minimise handling time and ensure fish welfare, as the ultrasound procedure was time‐consuming and, given the size of the fish and the probe's field of view, only one lobe could be reliably imaged at each sampling event. For standardisation, we consistently measured the left lobe and assume bilateral symmetry between ovarian lobes, following the approach of McGarvey et al. (2021). Ovary lobe volume was calculated from the combination of six geometrical shapes: four trunked cones of ellipsoid base (representing ovary sliced sections) and two cones of ellipsoid base (ovary ends). The volume of the geometrical shapes was calculated from 11 different measurements taken on the ovary transversal plane: the ovary length (measured with a tape on the fish flank, after locating the ovarian ends by ultrasound) and two cross‐sectional (height and width) measurements (Figure 1a) at five equidistant points along the longitudinal axis of the ovary (Figure 1b). The device's calliper function was used for calculating the cross‐sectional measurements. Then, the ovary left lobe volume was duplicated to obtain the total ovary volume.

At each scheduled sampling date, all fish from a given cage were transferred from the sea cages to the laboratory's indoor facilities. Before ultrasound measurements, they were tranquillised with a phenoxyethanol dose of 600 ppm. Fish were held lying on their right side pointing downwards, with their left flank facing upwards, in a water pan with enough water to cover the entire body and a phenoxyethanol dose of 300 ppm to keep them sedated during the ultrasound examination. Body weight (to the nearest 5 g) and total length (to the nearest millimetre) of the fish were also measured.

Ultrasound examinations were performed using an ultrasound portable unit (Exapad, IMV Imaging) with a linear transducer (7.5–15 MHz). Ultrasound scanning of fish was performed at 7.5–10 MHz frequency, depth penetration was set between 50 and 70 mm with the focus point at 15–25 mm and a signal amplification (gain) was set between 80% and 90%.

### 2.3 Calibration

To calibrate and validate ultrasonography as a suitable method for estimating ovary weight in European seabass adults, the ovary volume as calculated from ultrasound measurements was regressed against the actual ovary weight. As mentioned earlier, a subsample of 36 specimens were euthanised sequentially throughout the experiment timeframe to ensure that the full gradient of ovary size was sampled. Because ultrasound examinations and necropsies are both time‐consuming procedures, it was not feasible to conduct them on the same day. Fish were therefore euthanised on the day immediately following their final ultrasound examination, under the assumption that ovary size would experience negligible variation within the <24‐h interval. Once euthanised, ovaries were dissected, measured and weighted (to the nearest 0.1 g). A simple linear model was considered, and the parameters of the regression line were estimated using the *lm* function of the R package (R Core Team, 2022).

### 2.4 Modelling ovary weight dynamics

After validation (see Section 3), ultrasound measurements of ovary volume allowed reliable estimation of the temporal changes in the ovary weight of each fish. Only fish with six or more ultrasound ovary measurements were used for modelling ovary weight dynamics, being a total of 32 of the 48 fish monitored. The temporal pattern of the expected ovary weight of the *i* fish at time *t* (*W*_*t,i*_) was adjusted to a normal function, defined by the maximum ovary size (*h*, in grams), the date at which *h* is attained (*μ*, in days from January 1) and the spread of the reproductive period (*s*, in days):

(1) $$W_{t,i}=h_{i}e^{-\frac{1}{2}\left(\frac{{\left(t-\mu_{i}\right)}^{2}}{s_{i}^{2}}\right)}$$

The ovary weight calculated from ultrasound measurements (*WU*_*t,i*_) was assumed to be normally distributed around *W*_*t,i*_ with a standard deviation (*σ*_*m*_), which accounts for the measurement error at the observation level:

(2) $${\mathrm{WU}}_{t,i}\sim \text{Normal}\left(W_{t,i},\sigma_{m}\right)$$

The expected value of *h* for the *i* fish, $\bar{h_{i}}$ is predicted from a linear combination of the feed pellets intake score (*intake*_*i*_) (Equation 5, where the parameter of interest is the slope, *α*_*1*_). Then, the *h*_*i*_ value that best fits Equation (3) is assumed to be a random realisation from a normal distribution with a mean resulting from the sum of $\bar{\mathrm{hi}}$ and a cage‐level random contribution (Equation 4):

(3) $$h_{i}\sim \text{Normal}\;\left(\bar{h_{i}}+h_{\text{cage}},\sigma_{h}\right)$$

(4) $$h_{\text{cage}}\sim \text{Normal}\;\left(0.0,\sigma_{h,\text{cage}}\right)$$

(5) $$\bar{h_{i}}=\alpha_{0}+\alpha_{1}\times {\text{intake}}_{i}$$

The three parameters describing the ovary dynamics (*h*, *s* and *μ*) were modelled using the same equations. Thus, here only those concerning *h* are detailed (intercepts and slopes in Equation 5 for *s* and *μ* are mentioned as *β*_*0*_, *β*_*1*_, *γ*_*0*_ and *γ*_*1*_, respectively, in Table 1). Model parameters were estimated using a Bayesian approach, implemented in a custom R (R Core Team, 2022) script using STAN (https://mc-stan.org/) throughout the *cmdrstan* library (Gabry et al., 2023). Three independent chains were run, and convergence was evaluated by visual inspection of the trace‐plots and the Gelman–Rubin statistic (Gelman & Rubin, 1992). Posterior distributions were estimated based on 10,000 iterations per chain after appropriate warm‐up (2000 iterations). Weakly informative priors were assumed for all parameters.

**TABLE 1:** STAN result values for the different estimated variables, including the median, 95% CI, r_hat values and ess_bulk values.

Variable	R code symbol	Q5	Median	Q95	R_hat	Ess_bulk
$\gamma_{0}$	gamma0	5.3081	27.84	52.3062	1.0001	10,340
$\gamma_{1}$	gamma1	−0.5272	0.4479	1.3918	1.0001	12,149
$\sigma_{\mu }$	mu_sd	12.6954	17.2254	23.5562	1.0002	12,500
$\sigma_{\mu ,\text{cage}}$	mu_cage_sd	0.5424	6.0260	20.1076	1.0032	2169
$\beta_{0}$	beta0	28.1140	42.5744	57.7998	1.0004	7881
$\beta_{1}$	beta1	−0.8420	−0.2567	0.3210	1.0003	8495
$\sigma_{s}$	sigma_sd	5.1566	7.7083	11.4741	1.0002	8045
$\sigma_{s,\text{cage}}$	sigma_cage_sd	0.3450	3.2999	11.2304	1.0010	2356
$\alpha_{0}$	alpha0	−69.2916	51.3287	175.2542	1.0000	25,830
$\alpha_{1}$	alpha1	5.2909	11.1190	16.8114	1.0002	26,375
$\sigma_{h}$	h_sd	127.4980	163.5165	211.6990	1.0000	21,070
$\sigma_{h,\text{cage}}$	h_cage_sd	13.2549	75.8310	172.7523	1.0010	3962
$\sigma_{m}$	sd_within	47.7893	51.4406	55.6094	1.0001	24,333

### 2.5 Animal welfare statement

All animal care procedures were approved by the Ethical Committee of Animal Experimentation of the University of the Balearic Islands (CEEA‐UIB, ref. 153/12/20) and authorised by the Department of Environment, Agriculture and Fisheries of the Government of the Balearic Islands (ref. 2021/17/AEXP). All the procedures were carried out by trained competent personnel, in accordance with European Directive 2010/63/UE and Spanish Royal Decree RD53/2013 to ensure good practices for animal care, health and welfare.

## 3 RESULTS

### 3.1 Ultrasonography validation

**FIGURE 2:**
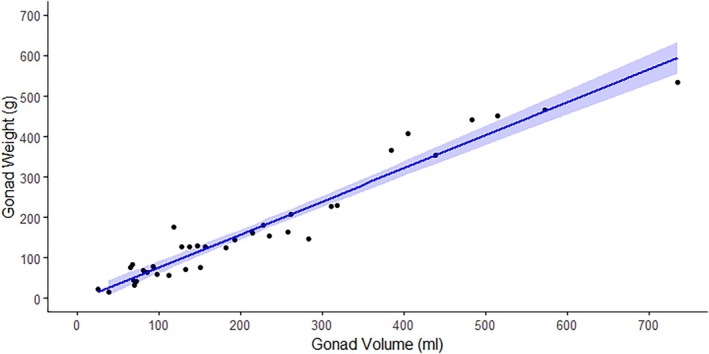
Relationship between the actual ovary weight and the ovary volume as calculated from ultrasound measurements.

The ovary volume as calculated from ultrasonographic measurements was highly correlated (*r* = 0.97) with the actual ovary weight (Figure 2). The slope of the linear regression was 0.82 (95% CI between 0.74 and 0.89), thus suggesting ovarian density is smaller than 1. Overall, this result strongly supports that ultrasonographic measurements can provide accurate and precise estimates of real time in vivo ovary weight.

### 3.2 Ovary weight dynamics

**FIGURE 3:**
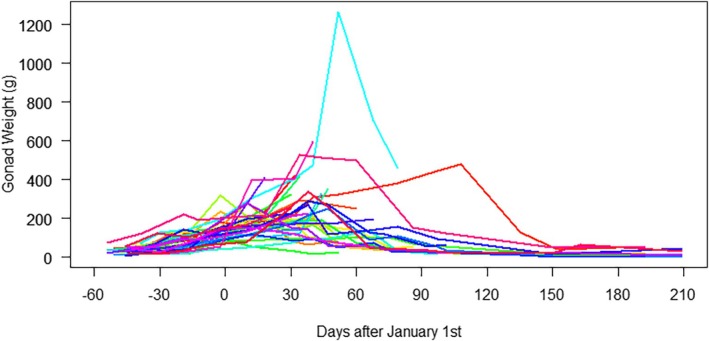
Temporal dynamics of ovary weight for the 32 fish analysed with six or more ultrasound measurements. Each colour of the spectrum represents an individual fish. The large between‐fish differences stand out over a general reproductive peak at winter.

The temporal dynamics of ovary weight of 32 females with at least six sequential ultrasound measurements are displayed in Figure 3. Over a general pattern of spawning during winter, fish exhibited marked individual differences in the maximum ovary size, the date at which it is attained and the spread of the ovarian growth and depletion. The magnitude of between‐individual variability is confirmed after fitting the ovary weight calculated from the ovary volume to the model in Equations ((1), (2), (3), (4), (5))–((1), (2), (3), (4), (5)). The estimated maximum ovary size (*h*) ranged between 56.4 and 1048.9 g (a 18.6‐fold difference). The date at which *h* is attained (*μ*) ranged between 14.1 and 78.7 days after 1 January (15 January and 20 March). Finally, the spread of the reproductive period expressed as the standard deviation of the Gaussian function describing the ovarian cycle (*s*) ranged between 16.8 and 44.7 days.

**FIGURE 4:**
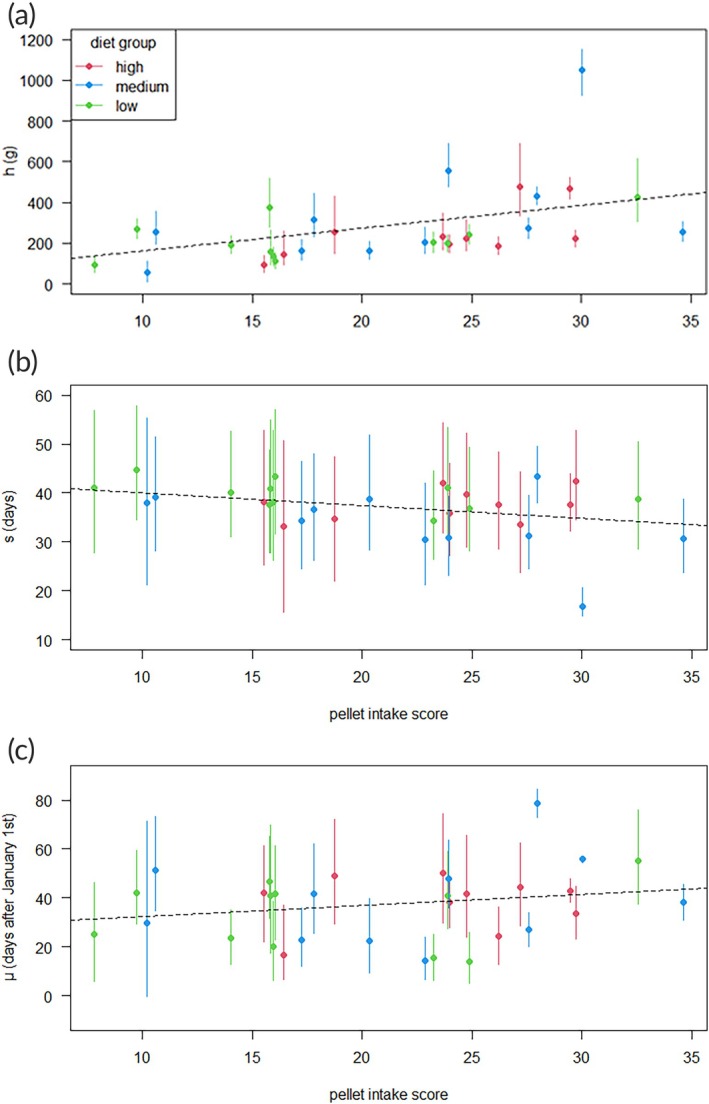
Relationship between the parameters describing ovary weight dynamics at the fish level and standardised feed intake [a: Maximum ovary size, b: Spread of the reproductive period, c: Date at which maximum ovary size (*h*) is attained]. Different colours represent different diet groups. The dotted line indicates the slope of the linear relationship between intake and each parameter. Each dot represents an individual fish value for the given parameter and pellet intake score, with whiskers indicating the 95% CI.

The estimated values of *h*, *s* and *μ* for each fish are plotted against the feed intake of the fish at Figure 4. The average standardised pellet consumption (*intake*_*i*_) is highly variable at the fish level (between 4.9 and 56.0 pellets per meal, with each feed pellet weighing 0.888 ± 0.066 g). Note that the variability between the fish that have been fed with the same diet is clearly larger than the between‐diet differences (Figure 4: 18.4 ± 7.5 pellets per meal for the *low*, 22.4 ± 9.4 for the *medium* and 26.5 ± 11.2 for the *high* diet). For instance, in the case of the *high* diet (fish receiving 90% of the feed amount recommended by the producer), the difference in consumption between the fish that consumes the least and the fish that consumes the most is 15.5 and 56.0 pellets per meal, respectively (a 3.6‐fold difference). This difference increases as the food availability decreases [4.5‐fold difference in *medium* (values between 10.2 and 46.0) and 6.6‐fold difference in *low* diet (values between 4.9 and 32.5)]. This fact strongly suggests that a small proportion of fish in each cage are hoarding most of the available food, whereas others may be consuming less than their nutritional requirements.

As expected, these differences on feed consumption are affecting the ovary weight. Specifically, the estimated maximum ovary weight (*h*) is linearly dependent on the fish‐specific *intake* score [slope (α_1_ in Table 1) = 11.1 ovary g/pellets per meal; 95% CI between 5.3 and 16.8]. Conversely, it seems that the *intake* score is not affecting either the date at which *h* is attained (*μ*) or the spread of the ovarian cycle (*s*), as the confidence intervals (CI) for the respective slopes include the zero. The estimates and the CI for all the parameters of the model in Equations ((1), (2), (3), (4), (5))–((1), (2), (3), (4), (5)) are detailed in Table 1.

**FIGURE 5:**
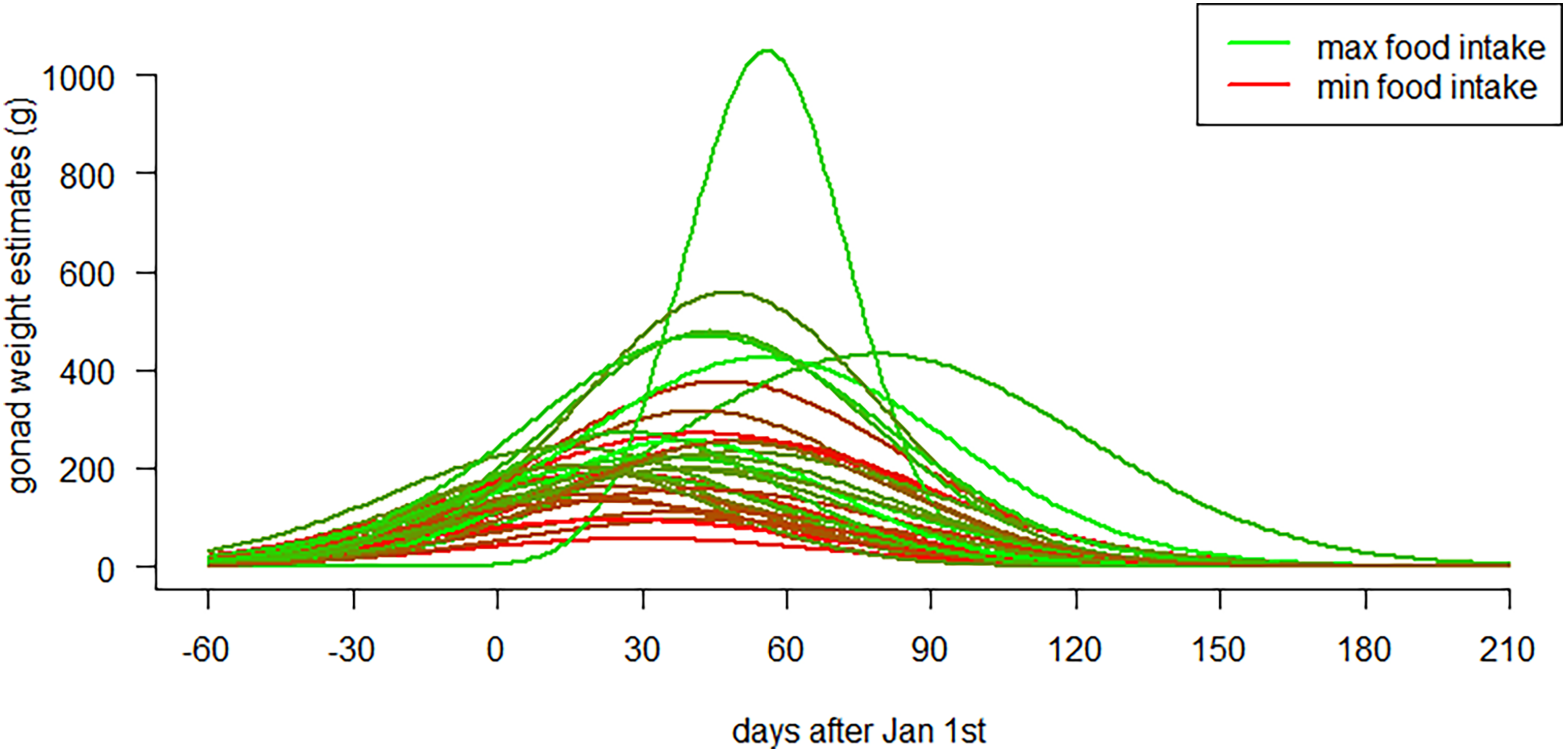
Estimated ovary weight dynamic at the fish level. The colour ramp between green and red is proportional to feed intake.

The estimated ovary weight dynamics for the 32 fish with at least six sequential ultrasound measurements are presented in Figure 5. Individual estimates for all 32 fish (and *intake*_*i*_ scores), plotted against ovary weight measurements, are provided in Supplementary Materials in Data S1. Again, the between‐fish variability is apparent, but is also self‐evident that fish with larger feed intakes tend to develop larger ovaries. The fish‐level values of *h*, *μ* and *s*, as well as fish‐level plots comparing the expected ovary weight according to the model in Equations ((1), (2), (3), (4), (5))–((1), (2), (3), (4), (5)) and the ovary weight as calculated from the observed ultrasonographic measurements are reported in Supplementary Materials in Data S1, as well as individual estimates of *h*, *μ* and *s* and the individual intake scores of all 32 fish.

## 4 DISCUSSION

The results confirm that ultrasonography is an accurate and reliable approach for non‐invasive, in vivo assessment of ovary size in adult female *D. labrax*. This is evidenced by the high correlation between the ovary volume calculated from ultrasound measurements and the actual ovary weight. Despite some differences between ultrasound measurements and the actual ovary size observed, the calibration function provided here, based on actual weighed ovaries and covering a wide range of sizes, produces precise estimates of ovary weight.

The handling protocol for ultrasound examinations has been proved to be safe and represents minimal risk to the fish welfare, which exhibited a normal ovarian development throughout the whole reproductive cycle. Moreover, the protocol allows the efficient longitudinal monitoring of a relatively large number of individuals throughout the reproductive season. In summary, ultrasonography can be used for an effective monitoring of ovary size dynamics in European seabass, providing real‐time insights for broodstock management with minimal harm to the fish. These advantages are clearly worth the investment in equipment and training (Webb et al., 2019).

Ultrasonography has been previously used to assess gonadal traits in fish. Macrì et al. (2013) examined juvenile *D. labrax* with the main objective of developing a non‐invasive method for sex identification. However, the results from that study (juveniles) are not directly comparable to the results reported here (4‐year‐old adults). Jennings et al. (2005) performed a conceptually similar study on adult *Morone saxatilis*, a species belonging to the same taxonomic family as *D. labrax*. Their approach also involved calibration of ultrasound‐derived measurements against actual gonad weights, although they used a logarithmic regression model based on mean gonad area from five cross‐sectional images, whereas we applied a linear calibration using six images and 11 linear measurements. Despite these methodological differences, both studies yielded comparable coefficients of determination (adjusted *r* ^2^), supporting the robustness of ultrasonography as a quantitative tool for estimating gonad size in large teleosts.

After a successful calibration, ultrasonography was used here to describe the ovarian development of 48 European seabass females. Although it is well known that this species spawns in winter (Pawson et al., 2007), we are reporting for the first time the marked between‐fish variability over this general trend, as depicted by ovary weight directly calculated from ultrasound measurements (Figure 3) and estimated ovary weight dynamics resulting from fitting these observations to a Gaussian function (Figure 5). Individual fish exhibit specific ovarian development patterns that ultimately may reflect different strategies for the spawning timing and for the ovary growth and depletion pace.

The ovary weight calculated from ultrasonography measurements is well explained by the model in Equations ((1), (2), (3), (4), (5))–((1), (2), (3), (4), (5)), which describes ovary weight dynamics with a unimodal and symmetric temporal trend, characterised by the maximum ovary weight (*h*), the date at which *h* is attained (*μ*) and the temporal spread of the ovarian cycle (*s*). The largest estimated ovary weight was 18.6‐fold larger than the smallest one. It is worth noting that these two fish belonged to the same cage (*medium* diet group), suggesting the existence of interindividual differences in reproductive capability, even among fish of the same age and similar initial size, reared under identical conditions of temperature and photoperiod. In spite of its conceptual simplicity, the good fit of the data to the model at the fish level (Supplementary Material in Data S1) and its apparent predictive power, we recognise that this model is not mechanistic. Asymmetric patterns of ovarian lobes growth and depletion paces could also be considered.

Assuming that ovary maximum weight could be a proxy of the reproductive potential, the results obtained would support that higher feed intakes lead to greater reproductive potentials, as already suggested by Serrat et al. (2019).

Despite fish in each cage have been fed at three contrasted diet levels (see Section 2.1), it is noteworthy that the feed amount ultimately consumed by a fish depends more on its individual capability, rather than on the total feed supplied at cage level. It is apparent that a small proportion of fish in each cage hoarded most of the available feed, whereas others consumed far less than their optimal nutritional requirements. Accordingly, the model in Equations ((1), (2), (3), (4), (5))–((1), (2), (3), (4), (5)) was explicitly designed to test the existence of a linear effect of food intake on each one of the three parameters of ovarian dynamics. Strong evidence was found (Table 1) for feed intake affecting maximum ovary weight, but not *μ* and *s*, which seem unrelated with feed intake. Interestingly, cage‐level variability was small (Table 1), reinforcing the relevance of individual feed consumption over total feed supplied at cage‐level. However, in aquaculture, despite precise control over feed servings at pen level, individual fish largely vary in their food consumption if not fed ad libitum. Because of this inherent variability, fish do not consume the same average amount of food, which is proposed as the main cause of sorting issues (Campeas et al., 2009; Ryer & Olla, 1996).

Certainly, the maximum ovary weight is also well correlated with fish size. Still, we recognise that the linear relationship proposed in Equations ((1), (2), (3), (4), (5))–((1), (2), (3), (4), (5)) is an oversimplification. The vast number of processes implied in the energy flow from feed to ovarian tissues will be better addressed by bioenergetic models. In this sense, the DEB theory (Kooijman, 2010) may improve our understanding of the relationship between weight and feed intake. Particularly, DEB theory proposes that a fixed ratio of the energy assimilated is invested in reproduction. Therefore, between‐fish differences in this ratio will result in different life‐history strategies, with some promoting faster somatic growth and others promoting a larger energy investment in reproductive processes. Between‐fish differences in the efficiency of processes related to food acquisition and/or processing, as well as in somatic maintenance costs, may also affect the available energetic outcome for growth and reproduction. The combination of all these processes will probably modulate the clear but linear relationship between food intake and ovary size reported here.

Specific reproductive modules within the DEB model have been proposed to predict not only fecundity but also the temporal dynamics of reproduction (Muller et al., 2019; Pecquerie et al., 2009). Ultrasonography, when combined with other data such as blood levels of sexual hormones and yolk egg precursors, or gonad histological data, can enhance the predictive capability of DEB and other bioenergetic models. This, in turn, may allow us to design selection programmes with a mechanistic basis. Ultimately, ultrasonography can be exploited to select phenotypes with smaller reproductive energy investments or early/late spawners.

In summary, ultrasonography is a promising technique for measuring ovary size and assessing ovarian maturation and the spawning process at the individual level. It enables real‐time and continuous monitoring of a large number of fish, helping to describe individual‐specific reproductive patterns (Bureau du Colombier et al., 2015). Unlike other methods such as gonad histology, ultrasonography is able to assess reproductive condition indices without killing specimens (Blythe et al., 1994; McGarvey et al., 2021). The observed variability in ovarian development patterns among fish suggests that individuals of the same species may respond differently to dietary and environmental factors. This detailed temporal analysis highlights the importance of considering interindividual variability when assessing reproductive processes. Consequently, ultrasonography is supported as a valuable tool for a non‐invasive monitoring of ovarian dynamics in *D. labrax*, as well as other fish species, throughout their life span.

## AUTHOR CONTRIBUTIONS

**Joaquim Tomàs‐Ferrer**: conceptualisation, data curation, formal analysis, investigation, methodology, resources, visualisation, writing – original draft. **Irene Moro‐Martínez**: formal analysis, visualisation, writing – review and editing. **Enrique Massutí‐Pascual**: data curation. **Amàlia Grau**: conceptualisation, funding acquisition, methodology, project administration, resources, supervision, writing – review and editing. **Miquel Palmer**: conceptualisation, formal analysis, funding acquisition, methodology, project administration, software, resources, supervision, visualisation, writing – review and editing.

## FUNDING INFORMATION

Joaquim Tomàs‐Ferrer and Irene Moro‐Martínez were supported by a Ph.D. fellowship (FPI) from the Instituto Nacional de Investigación y Tecnología Agraria y Alimentaria (INIA) (FPI‐INIA‐2019 PRE2019‐091411 and FPI‐INIA‐2020 PRE2020‐093901, respectively). This is a contribution of the Joint Research Unit IMEDEA‐LIMIA. This project received financing from METARAOR project from the Spanish Government (PID2022‐139349OB‐I00).

## CONFLICT OF INTEREST STATEMENT

The authors declare that they have no competing interests.

## Supporting information


**DATA S1.** Supporting information.

## Data Availability

Both the data and the model script are provided at the GitHub repository (https://github.com/JTomasFerrer/US_fish_gonad_dynamics_JTFetal2024) to adhere to data availability policies and encourage reproducibility.
