# Supplementary material for: Ultrasonography as a non‐invasive technique to assess the effects of diet on the ovaries of female European seabass (Dicentrarchus labrax)
Source: J Fish Biol. 2026 Apr 15;109(1):531–41. doi: 10.1111/jfb.70406 (PMC13397255; doi:10.1111/jfb.70406)
Supplement: Supplementary file 1 — DATA S1. Supporting information. [file JFB-109-531-s001.zip › Tomàs-Ferrer_etal_US_figure_captions_sup_mat_rev.docx]

# Figures in supplementary material

Figure I: Comparison between ovary weight ultrasound measurements (dots) and the Gaussian estimates for ovary weight (lines) for the 32 fish analysed with 6 or more ultrasound measurements. Each fish’s intake score is also indicated.
