# Supplementary material for: Ultrasonography as a non‐invasive technique to assess the effects of diet on the ovaries of female European seabass (Dicentrarchus labrax)
Source: J Fish Biol. 2026 Apr 15;109(1):531–41. doi: 10.1111/jfb.70406 (PMC13397255; doi:10.1111/jfb.70406)
Supplement: Supplementary file 1 — DATA S1. Supporting information. [file JFB-109-531-s001.zip › Tomàs-Ferrer_etal_US_sup_mat_tables_rev.docx]

Table I: median and 5% and 95% percentiles for the 3 estimated ovarian dynamics parameter values (h, μ and s) for all the 32 fishes analysed.

| fish |  | h |  |  | μ |  |  | s |  |
| --- | --- | --- | --- | --- | --- | --- | --- | --- | --- |
| code | p5 | median | p95 | p5 | median | p95 | p5 | median | p95 |
| BB1 | 278.3 | 375.7 | 519.3 | 31.6 | 46.5 | 65.1 | 27.8 | 37.6 | 48.7 |
| BB2 | 388.2 | 431.6 | 478.0 | 72.9 | 78.7 | 84.4 | 38.1 | 43.4 | 49.5 |
| BB4 | 224.8 | 270.6 | 318.9 | 29.5 | 41.9 | 59.3 | 34.4 | 44.7 | 57.8 |
| BB6 | 92.2 | 144.8 | 259.2 | 6.6 | 16.7 | 37.0 | 15.6 | 33.2 | 50.7 |
| BN1 | 155.4 | 202.9 | 256.4 | 6.3 | 15.7 | 25.1 | 26.5 | 34.4 | 44.6 |
| BN4 | 146.9 | 189.9 | 236.8 | 12.8 | 23.5 | 35.3 | 31.1 | 40.2 | 52.5 |
| BN5 | 119.7 | 161.8 | 208.5 | 9.5 | 22.5 | 39.6 | 28.3 | 38.6 | 51.9 |
| BV1 | 195.6 | 241.9 | 293.2 | 5.2 | 14.1 | 25.8 | 28.2 | 36.7 | 49.2 |
| BV2 | 115.5 | 162.3 | 217.8 | 12.0 | 22.9 | 35.9 | 24.4 | 34.3 | 46.5 |
| BV4 | 74.9 | 112.0 | 152.0 | 22.7 | 41.7 | 61.3 | 31.7 | 43.3 | 57.1 |
| BV5 | 229.8 | 316.5 | 446.1 | 25.3 | 41.7 | 62.3 | 26.3 | 36.5 | 48.0 |
| NB2 | 11.0 | 56.4 | 110.8 | -0.4 | 29.7 | 71.5 | 21.2 | 38.0 | 55.2 |
| NB3 | 142.9 | 186.2 | 233.3 | 12.8 | 24.3 | 36.2 | 28.6 | 37.5 | 48.4 |
| NB4 | 305.4 | 424.3 | 614.0 | 37.6 | 55.2 | 76.0 | 28.6 | 38.8 | 50.6 |
| NB5 | 194.8 | 257.0 | 357.8 | 34.8 | 51.4 | 73.3 | 28.2 | 39.0 | 51.4 |
| NB6 | 55.1 | 95.5 | 140.5 | 22.0 | 42.0 | 61.4 | 25.2 | 38.1 | 52.8 |
| NV2 | 924.4 | 1048.9 | 1153.2 | 54.6 | 55.8 | 57.0 | 14.9 | 16.8 | 20.7 |
| NV3 | 168.0 | 232.6 | 349.7 | 29.9 | 50.1 | 74.5 | 31.7 | 41.9 | 54.3 |
| NV4 | 93.0 | 156.5 | 262.4 | 17.4 | 41.1 | 69.6 | 27.7 | 40.8 | 55.0 |
| NV6 | 152.7 | 196.6 | 243.5 | 27.8 | 38.2 | 48.4 | 27.1 | 35.8 | 46.1 |
| VB1 | 56.1 | 94.4 | 138.0 | 5.9 | 25.3 | 46.4 | 27.7 | 41.1 | 56.9 |
| VB5 | 208.1 | 256.5 | 307.4 | 30.9 | 38.2 | 45.7 | 23.8 | 30.7 | 38.8 |
| VB6 | 181.2 | 221.5 | 263.6 | 23.2 | 33.6 | 44.7 | 34.6 | 42.5 | 52.8 |
| VN1 | 156.8 | 198.7 | 243.5 | 27.4 | 41.0 | 59.2 | 31.5 | 41.1 | 53.4 |
| VN3 | 332.7 | 477.6 | 691.3 | 28.4 | 44.4 | 62.5 | 23.6 | 33.6 | 44.3 |
| VN5 | 147.8 | 203.4 | 276.8 | 6.6 | 14.5 | 24.0 | 21.1 | 30.5 | 42.1 |
| VN6 | 164.5 | 224.5 | 316.7 | 23.9 | 41.8 | 65.7 | 28.9 | 39.8 | 52.2 |
| VV1 | 90.9 | 133.3 | 182.2 | 6.2 | 20.3 | 38.6 | 26.3 | 38.1 | 52.9 |
| VV2 | 476.2 | 557.1 | 690.2 | 35.7 | 47.9 | 63.7 | 23.2 | 30.8 | 39.3 |
| VV3 | 417.7 | 469.2 | 523.9 | 38.0 | 43.0 | 47.9 | 32.1 | 37.6 | 44.0 |
| VV5 | 222.2 | 272.7 | 326.2 | 20.1 | 27.1 | 34.0 | 24.4 | 31.3 | 39.4 |
| VV6 | 147.6 | 253.3 | 431.6 | 29.5 | 49.2 | 72.0 | 22.0 | 34.6 | 47.3 |
