# Supplementary figures and images for: Ultrasonography as a non‐invasive technique to assess the effects of diet on the ovaries of female European seabass (Dicentrarchus labrax)
Source: J Fish Biol. 2026 Apr 15;109(1):531–41. doi: 10.1111/jfb.70406 (PMC13397255; doi:10.1111/jfb.70406)

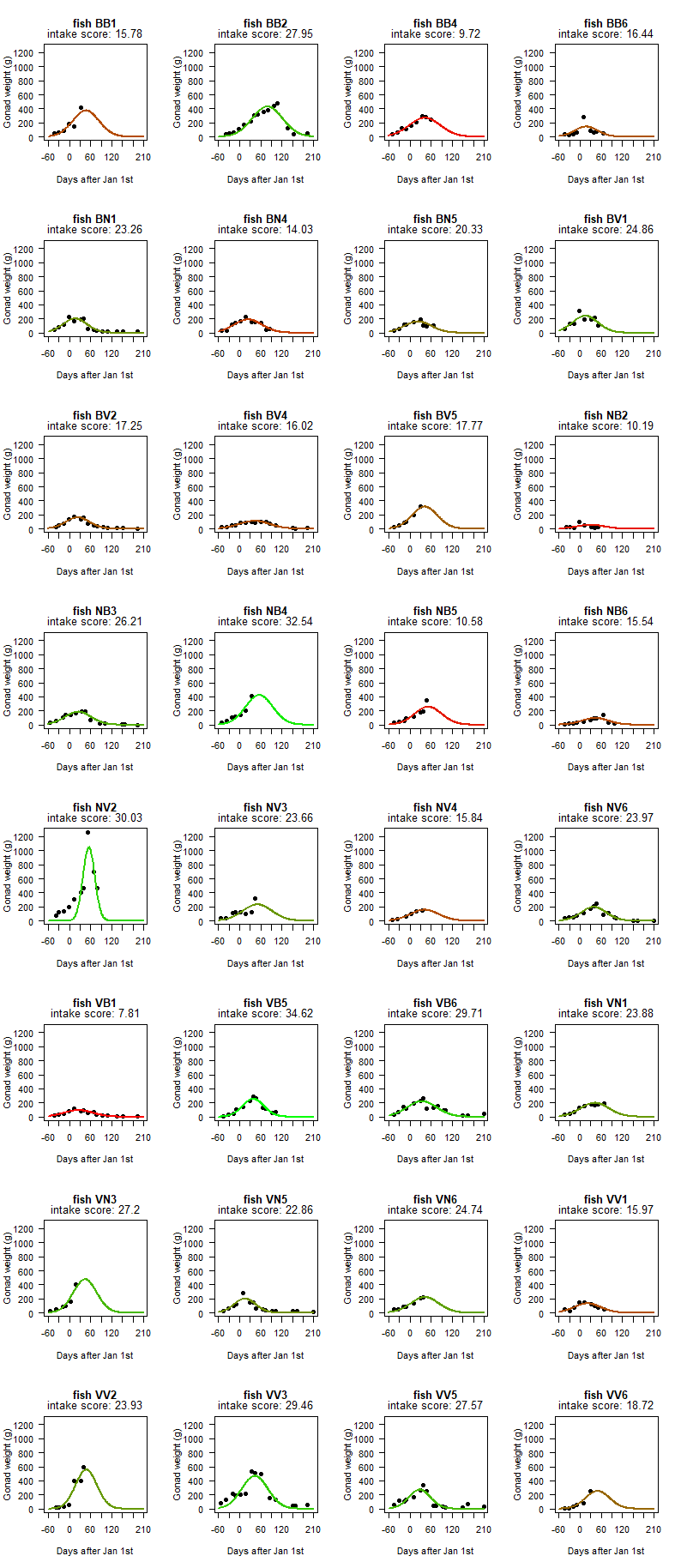

Supplement: Supplementary file 1 — DATA S1. Supporting information. [file JFB-109-531-s001.zip › SM_Figure_1.png]
